# The clinical management of testosterone replacement therapy in postmenopausal women with hypoactive sexual desire disorder: a review

**DOI:** 10.1038/s41443-022-00613-0

**Published:** 2022-10-05

**Authors:** Maria Uloko, Farah Rahman, Leah Ibrahim Puri, Rachel S. Rubin

**Affiliations:** 1 Department of Urology, University of California, San Diego, CA USA; 2 Loyola Stritch School of Medicine, Chicago, IL USA; 3 Department of Urology, University of California, San Francisco, CA USA; 4 Department of Urology, Georgetown University, Washington, DC USA

**Keywords:** Hormonal therapies, Diagnosis

## Abstract

As women age, there is an overall decrease in androgen production due to decline of ovarian and adrenal function during menopause. Androgens have been demonstrated to play an important role in sexual motivation in women. As a result, many postmenopausal women experience Female Sexual Dysfunction (FSD) which are a group of disorders that pertain to sexual arousal, desire, orgasm, and pain. A prevalent manifestation of FSD is Hypoactive Sexual Desire Disorder (HSDD) or the absence of sexual fantasies, thoughts, and/or desire for or receptivity to sexual activity. There is gaining interest in the use of Testosterone Replacement Therapy (TRT) for the treatment of HSDD in postmenopausal women. This article reviews the literature on the relationship of androgen decline and HSDD, describes our methodology for evaluation, diagnosis of HSDD, and the use of TRT in treating postmenopausal women with HSDD. Our results conclude that testosterone is a vital hormone in women in maintaining sexual health and function. TRT is an effective treatment option for postmenopausal people with HSDD. There is still limited data on the effectiveness in premenopausal people with HSDD. Further research in the strengths and weaknesses for the long-term effect of TRT in women of all ages is needed.

## Introduction

Androgens are important hormones that assist in the regulation and maintenance of the vulvovaginal complex, pelvic floor, bladder, and urethra as well as vital sexual functions including vaginal lubrication [1–4]. Contrary to popular belief, androgen production serves a greater importance than just estrogen production in women [5]. Circulating testosterone is measured in nanomolar concentrations (ng/dl) in women, an order 1000 times more than circulating estradiol which is measured in picomolar concentrations (pg/dl) [5, 6].

**Fig. 1:**
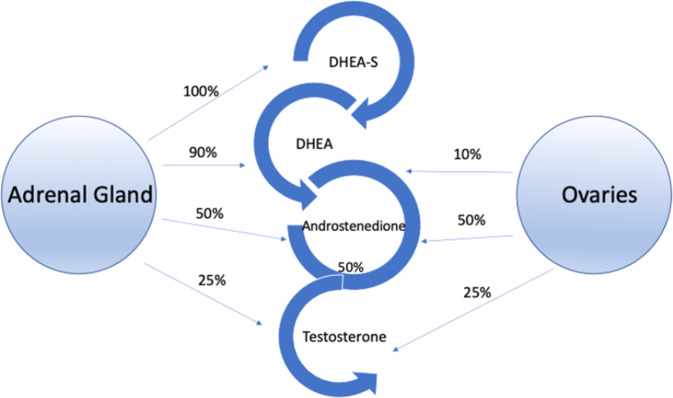
Overview of androgen synthesis in the premenopausal ovary and adrenal gland. Overview of the origin of androgen synthesis in premenopausal women.

Testosterone is produced by the ovaries and adrenal glands in women (Fig. 1) [7, 8]. Of the androgens, only testosterone and dihydrotestosterone (DHT) can bind the androgen receptors (AR) [9]. Testosterone travels through circulation predominately bound to sex hormone binding globulin (SHBG) with high affinity (~65%) and albumin with less affinity (~30–45%) than SHBG [10, 11]. There is an inverse relationship between SHBG levels and free testosterone, the higher the levels of SHBG the lower the level of bioavailable free testosterone. SHBG production can be affected by various conditions (see Table 1) [10]. Once testosterone is unbound from albumin or SHBG, it can bind to the AR as free testosterone or be converted to the more potent DHT which has a stronger affinity to the AR [9]. Once bound to the receptor, it is translocated into the nucleus for gene transcription [12]. This results in gene activation of various cellular activity including metabolism, cognition, and sexual function [13]. As women age, there is an overall decrease in androgen production due to age-related decline of ovarian and adrenal function, which occurs during menopause [14]. The steady decline of testosterone begins after early reproductive years with a small increase during menopause due to the decrease in SHGB [15]. In comparison, women who undergo surgical menopause have a marked and permanent decrease in testosterone production [16]. Their levels are up to 50% lower than women who undergo menopause naturally [16].

**Table 1:** Factors that increase or decrease levels of SHBG in women.

Factors that increase sex hormone binding gobulin	Factors that decrease sex hormone binding gobulin
Aging	Obesity
Infectious disease (HIV, Hepatitis C)	Diabetes, insulin resistance
Hyperthyrodism	Hypothyroidism
Hepatocellular dysfunction	Polycystic ovarian syndrome
Elevated estrogen	Hyperprolactinaemia
Medications (thiazolidines, anticonvulsants, oral contraceptives, selective estrogen receptor modulators)	Medications (glucocorticoids, tyrosine kinase, inhibitors, androgens)
Sex hormone binding polymorphisms	Hypercorticism
Extreme weight loss	Acromegaly
	Nephrotic syndrome

Androgens have been demonstrated to play a role in sexual motivation in women, including but not limited to exhibiting sexual interest, initiating sexual activity, and responding to sexual stimulation [17–22]. As a result, decreased testosterone levels have been hypothesized to be an important and reversible cause of HSDD. In 2019, The Global Consensus Position Statement on the Use of Testosterone Therapy for Women [23] endorsed the use of TRT in postmenopausal women with HSDD. This recommendation has been widely accepted and endorsed by ten international societies as the best evidence-based guidance to date on TRT in women. In 2021, the International Society for the Study of Women’s Sexual Health (ISSWSH) then published the first clinical practice guidelines on the use of TRT for the treatment of HSDD in menopausal women [24].

The present article aims to highlight the use of TRT in the management of the postmenopausal woman experiencing symptoms of HSDD. The review begins by providing a concise definition of HSDD under the DSM-IV and the evaluation and diagnosis of HSDD. It then describes the clinical use of testosterone in the setting of HSDD, followed by exploring various testosterone formulations, and monitoring in patients who continue to be on TRT. Finally, we explore future areas of research to illustrate strengths, weakness, opportunities and downfalls of the use of testosterone to improve sexual health and function in women experiencing HSDD.

## Female sexual dysfunction

FSD encompasses a group of disorders that address arousal, desire, orgasm, and pain [25]. It is classified in the DSM-IV as six disorders including: HSDD, Female Arousal Disorder (FAD), Female Orgasmic Disorder, Dyspareunia, and Vaginismus [26]. The revised DSM-V merged HSDD and FAD into Female Sexual Interest/Arousal Disorder (FSIAD). This new definition is controversial among experts in sexual medicine, as it decreases diagnostic accuracy [24, 27, 28]. The International Consultation on Sexual Medicine (ICSM) and The International Society for the Study of Women’s Sexual Health (ISSWSH) recommend that HSDD should be maintained as an entity separate from FSIAD, as previous studies have measured these as two separate outcome endpoints [28, 29].

HSDD is defined as “the absence of sexual fantasies and thoughts, and/or desire for or receptivity to, sexual activity that causes the personal distress or difficulties in their relationship lasting for at least 6 months” [26, 30, 31]. The cause of HSDD is often multifactorial and can include central processes, e.g., neuroendocrine imbalance, medication, hypogonadism, psychological distress, and cultural factors. e.g., religious, or cultural emphasis on sexual purity [31–33]. HSDD is associated with profound negative effects on mood, self-esteem, and partner relationships and can cause significant decrease in quality of life [33]. Nearly half of menopausal or postmenopausal women (ages 57–85) in the United States have some element of FSD with HSDD being the most reported form [33].This number is likely to increase in the upcoming years due to the progressively aging population [34]. It is estimated by 2030, around 1.2 billion will reach menopause, with an increasing number of people experiencing early menopause, now known as premature ovarian failure [35].

## Evaluation and diagnosis of HSDD

A biopsychosocial approach is recommended for optimal diagnosis and treatment of HSDD. This can include a mental health provider, sexual health medical expert, and/or physical therapist. A comprehensive history should be obtained in these patients including past medical, surgical, social, and sexual/relationship history. Primary goals of the biopsychosocial assessment are to appropriately diagnose the subtype of HSDD while ruling out reversible causes including depression, medication side effects, relationship issues, neurologic conditions, and dyspareunia. Validated questionnaires are a useful tool in the diagnosis of HSDD. The Female Sexual

Function Index (FSFI) [36] is a validated questionnaire [30, 37–39] used to assess 6 domains within sexual function in women. Other useful questionnaires for screening of HSDD include the

Sexual Interest and Desire Inventory [40], the Female Sexual Distress Scale [39], and the Decreased Sexual Desire Screener [37].

A comprehensive physical exam should be performed including a detailed genital exam, examining for signs of hypogonadism like decreased clitoral size, labial resorption, vestibular erythema or pallor, degree of vaginal atrophy, and elevated vaginal pH. A complete hormonal panel should be obtained including total testosterone, free testosterone, estradiol, and SHBG. Estradiol, prolactin, and thyroid-stimulating hormone can also be ordered if indicated [27]. Due to the low levels of testosterone levels in women compared to men, there are limitations in precision in the available assays used to measure testosterone. Radioimmunoassay (RIA) is the most used technique to measure total testosterone in women [41]. This assay is often unreliable in women, as it has not been standardized to measure testosterone levels in women nationally or internationally [42, 43]. If available, liquid chromatography (LC)/gas chromatography (GC) and LC-tandem mass spectrometry (MS/MS) assays should be used instead to measure total testosterone, as it provides a more reliable and accurate test [24, 42, 44–46]. The reference range of normal testosterone in women is provided. (Table 2).

**Table 2:** Reference ranges of testosterone level in women based on age.

Ages	Reference range of testosterone production by ovaries and adrenal glands (ng/dL)
20–29 years	45.5–57.5 ng/dL
20–39 years	27.6–39.8 ng/dL
40–49 years	27.0–38.6 ng/dL

Although there is no absolute testosterone value associated with HSDD [4, 33, 47], it is recommended to not prescribe testosterone to menopausal women with signs of elevated androgens (androgenic alopecia, hirsutism, acne, etc) or testosterone levels in the mid or high ranges [24]. Patients with low testosterone in the setting of elevated SHBG or on antiandrogen therapy (spironolactone, finasteride, etc.) should be counseled on the risk that they may have muted benefits from therapy. Initiation of testosterone can still be attempted in patients with nonmodifiable causes of elevated SHBG [48].

## Evidence for testosterone in HSDD

Testosterone has been shown to be effective alone or with used in conjunction with estrogen in postmenopausal women. A 2005 study by Buster et al. [49], which randomized 533 women into testosterone versus placebo found that testosterone increased total satisfying sexual activity as well as libido in comparison with placebo (1.56 episodes vs 0.73 episodes per 4 weeks). In a 2008 study by Davis et al. [50] 814 menopausal women (natural or surgical) with concurrent HSDD were randomized to either placebo group or treatment arms over 24 weeks. The treatment arms included subjects receiving testosterone at the doses of 150 μg/day or 300 μg/day via the patch. Participants in the 300 μg group had significantly greater Satisfying Sexual Events (SSEs) over 4 weeks when compared to the placebo group (episodes per 4 weeks: an increase of 2.1 episodes vs. 0.7, *P* < 0.001). This effect, however, was not seen in the patients randomized to the 150 μg/day group (1.2 episodes vs. 0.7, *P* = 0.11). In the ADORE study, Panay et al. [51] found similar results in their 2010 placebo-controlled, double-blind trial conducted over six months. 272 menopausal women were randomized to either receive a transdermal testosterone patch (300 μg/d) or a placebo. They found improvement in libido (*P* = 0.0007), SSEs, (*P* = 0.0089), and reduced personal distress (*P* = 0.0024) in the testosterone group compared with placebo. Regarding combination therapy, Simon et al. [52] found that testosterone administration (via a patch) in combination with estrogen improved libido and the frequency. This multicenter study of 562 women who underwent surgically induced menopause showed a total satisfying sexual activity from 2.82 to 4.92 episodes per 4 weeks in the treatment group in comparison to the placebo, 2.94–3.92 episodes (*P* = 0.0003) per 4 weeks measured by sexual activity log (SAL). In 2006, Shifren et al. [53] assessed the effects of testosterone (via a patch) in combination with estrogen ± progesterone in 549 women who had undergone natural menopause in a randomized placebo-controlled trial over the span of 24 weeks. Women randomized to the testosterone arm endorsed a statistically significant increase in the number of sexual events measured by SAL in comparison to placebo (placebo, 0.5 ± 0.23; testosterone, 2.1 ± 0.28, *P* < 0.0001 vs placebo).

Studies directly comparing the combination of estrogens/androgens have typically shown synergistic effects compared to estrogen or testosterone treatment alone. In 1985, Sherwin et al. [21] studied menopausal women that received the following: combination estradiol 8.5 mg and oral testosterone 150 mg versus estradiol 8.5 mg alone versus oral testosterone 150 mg alone versus placebo. They saw the greatest improvement in libido and arousal in the combined estrogen/androgen therapy. In a double-blinded randomized study by Huang et al. [54], 71 menopausal women (either surgical or natural) were observed after 12 weeks after being given a transdermal estrogen regimen. After 12 weeks of estrogen therapy, they were then randomized to receive weekly IM injections of either placebo, 3 mg, 6.25 mg, 12.5 mg, or 25 mg testosterone enanthate, respectively, for 24 weeks. There was found to be a dose-dependent improvement in sexual function. The greatest improvement was seen in the group receiving the highest dose of testosterone. Similar outcomes were found in the double-blind randomized trial by Lobo et al. [55], assessing 221 postmenopausal women given either oral combined esterified estrogen/methyltestosterone or oral esterified estrogen alone over 16 weeks. The combination group had improvement in scores. (3.3 ± 5.6 vs 1.3 ± 4.7; (*P* = 0.002).

Although transdermal testosterone is the recommended first-line option, there are studies to show the effectiveness of non-transdermal modes of delivery. Tungmunsakulchai et al. [56] compared the effects of placebo versus oral testosterone undecanoate 40 mg combined with oral estrogen in 70 postmenopausal women in a randomized double-blind study. The testosterone group had greater improvement seen in FSFI scores compared with placebo (FSFI scores: placebo, 28.6 ± 3.6; testosterone, 25.3 ± 6.7, *P* = 0.04 vs placebo). Raghunandan et al. [57] examined the effects of local vaginal estrogen with or without testosterone versus placebo (KY jelly) in 75 women who had undergone menopause either surgically or naturally with symptomatic vaginal atrophy and sexual dysfunction. Once randomized into their respective groups (estrogen ± testosterone versus placebo), study subjects then applied their respective dose twice weekly over 12 weeks. Outcomes were measured using the McCoy sexuality scores to compare prior and post-treatment effects. They found overall improvement from baseline in each treatment arm with the highest seen in the combination group of estrogen with testosterone. (Percentage improvement in McCoy score from placebo, estrogen-only, 42.4% *P* < 0.05 versus placebo; estrogen with testosterone, 147%, *P* < 0.01 versus placebo).

## Types of testosterone formulations

TRT has not been approved by the FDA for use in women with HSDD despite studies showing a positive effect. Clinicians should obtain informed consent before prescribing TRT due to the off-label indication for HSDD [24]. The consent should discuss shared decision-making between provider and patient based on the patient’s goals of care after detailed counseling regarding the benefits and risks of the off-label use of testosterone therapy in women [58, 59]. Patients should also be made aware that due to the off-label use, it is more cost-effective to pay “out of pocket” instead of using insurance which can result in higher costs to the patient [24]. Several formulations of testosterone require a minimum purchase (e.g., testosterone 1% gel requires 30 tubes minimum) but because the amount of testosterone required for women is significantly lower than men, the prescription lasts a pointedly long time. There are also significant costsaving options within the United States offering coupons such as GoodRx (www.goodrx.com).

There are several testosterone preparations currently FDA-approved for the treatment of hypogonadism in men. The route of administration can be buccal, nasal, subdermal, transdermal, or intramuscular (IM). Transdermal formulations (patch, gel, cream, spray) are the preferred and most used in women due to their ease of administration and the ability to titrate to physiological levels [24]. Patients should be counseled on the risk of transference of testosterone to other individuals and pets [60]. This risk can be reduced by application to a clean and dry area with little risk for transference like the back of the calf, the upper outer thigh, or the buttock, as well as diligent handwashing with soap and water immediately after application per FDA warning. The potential risk of long-term continual exposure through transference includes hair growth, virilization, and acne [60]. If contact does occur to individuals, it is recommended to wash the affected area with soap and water [60]. Adjustments in dosing are required when used in women, as the medication is formulated solely for use in men. The recommended dose adjustment is 1/10th the recommended starting dose in men [24]. For example, men prescribed 1% generic testosterone gel for hypogonadism would use one tube/day. The equivalent starting dose for women with HSDD would be one tube or packet every 10 days which equates to about 5 mg/day (0.5 ml). This can be up titrated to 10 mg/day (1.0 ml) as needed, pending laboratory assessment and symptom management [24]. Prescribers should specify resealable tubes to the pharmacy, as they are preferential to packets because they prevent evaporation which can change the testosterone concentration in the gel [24]. Compounded testosterone is not recommended for systemic TRT for the treatment of HSDD due to the inability to safely regulate the testosterone concentration within [23, 61, 62]. The recommended doses and formulations of testosterone are provided (Table 3).

**Table 3:** The recommended doses and formulations of testosterone by formulation, dose, frequency, and route of administration.

Formulation	Dose	Frequency	Route of administration
Testim 1%	1/10th tube, 5 mg	Daily	Transdermal
Axiron 2%	0.3 ml	Daily	Transdermal, underarm
Androgel 1%	1/10th tube, 5 mg	Daily	Transdermal
Testosterone Enthanate or Cypionate	100 mg/ml, inject 0.05 ml (5 mg)	Weekly	Intramuscular
Testosterone implant	75 mg, 1 pellet	Every 4–6 months	Subcutaneous

Other testosterone formulations can be considered but are not recommended as first-line therapies. IM injections can cause fluctuation and lead to supraphysiologic levels due to the slower metabolism. Oral preparations are typically not recommended due to fluctuations in levels of testosterone and the associated liver toxicity. Subcutaneous testosterone implants are not recommended, as there is no ability for dose titration and may result in supraphysiological levels [4, 63, 64].

## Monitoring

Each formulation of testosterone has different absorption rates altering the pharmacokinetics of its effect on the AR. Due to this variability, constant monitoring is key for safe and efficacious treatment. The goal of monitoring is to prevent excessive dosing and ensure appropriate dose titration [23, 24]. Prior to initiating treatment, baseline liver function testing and a fasting lipid panel should be obtained and monitored yearly. Of note, liver dysfunction and hyperlipidemia are contraindications for TRT. Total testosterone should be evaluated 3–6 weeks after initiation of TRT and repeated within 6 weeks anytime there is a dose adjustment during titration to physiologic levels as determined by the patient’s laboratory [24, 27, 29]. Patients should be counseled that it will take up to 12 weeks to see maximal results but to expect to see improvement in symptoms 6–8 weeks after initiation of treatment with some women seeing results as early as 4 weeks [65]. Qualitative and subjective treatment responses should be monitored, such as an increase in sexual desire, improvement in quality of life, and decrease in personal distress [59]. Self-reported validated instruments (e.g., FSFI, FSDS) can be administered as a supplemental and objective means to assess treatment outcomes [36,39]. Patients should also be assessed for side effects including acne, hirsutism, and androgenic alopecia. It is recommended that total testosterone does not markedly exceed the upper limit of the reference range to decrease the risk of adrenergic side effects [24]. These side effects can also occur with normal serum levels of total testosterone, usually as a result of low SHBG [66, 67]. Patients should be instructed to down titrate the amount of testosterone if they are found to have supraphysiologic levels of testosterone, even without signs or symptoms of androgenic excess. Labs should then be repeated after 2–3 weeks to ensure the resolution of supraphysiologic levels [23, 24].

Calculating free testosterone as well as obtaining SHBG can be helpful in patients that have total testosterone levels in the upper range of physiologic levels without symptomatic improvement in HSDD symptoms [23, 24]. There are several free bioavailable testosterone online calculators utilizing total testosterone and SHBG values [24]. Measurement of 5α-reductase should be considered in patients without clinical improvement in symptoms found to have normal total testosterone and free testosterone. In the scenarios of low free testosterone or decreased 5αreductase, experts have supported total testosterone levels higher than physiologic testosterone [68].

Once levels are stable, testosterone concentrations should be monitored every 4–6 months [47, 62]. If testosterone therapy results in improvement of HSDD, a drug holiday should be considered after 6–12 months of treatment to see if treatment is still required [24]. If there is a decline in symptoms after cessation of testosterone, therapy should then be resumed long-term to maintain the improvement in HSDD [24]. Testosterone treatment should be discontinued if there is no clinical improvement noted by the patient after 6 months [23, 47, 69].

## Future areas of research

In premenopausal women, the data is mixed regarding the efficacy of TRT. A cross-sectional study by Zheng et al. [70] of 588 premenopausal women examined the correlation of several domains of sexual health based on the Profile of Female Sexual Function (PFSF) questionnaire with androgen levels. They found a positive correlation in orgasm with levels of testosterone but found a weak correlation in sexual desire, arousal, and masturbation overall A cross-sectional study of 560 premenopausal women by Wåhlin-Jacobsen et al. [71] evaluated the correlation between the different androgens (total testosterone, free testosterone, androstenedione, and DHEA) and sexual desire using the FSFI. A positive correlation was seen with sexual desire and levels of free testosterone and androstenedione (*P* = 0.047 and 0.002, respectively) but not the other androgens. This presents further questions in the study design of using free testosterone as a more accurate endpoint compared to total testosterone, as it does not accurately reflect the amount of bioavailable testosterone. At this time, more studies are needed to fully evaluate the therapeutic use of testosterone in premenopausal women. Future areas of research should aim to utilize adequately powered, double-blind RCTs with standardized core outcomes, which are needed to systematically examine the use of testosterone therapy for women with FSD. Research to date has been limited by a lack of standardized laboratory methods for measuring testosterone, inhomogeneity in questionnaires used between studies, confounding outcomes in studies, selection bias, and lack of diversity in study participants. Many studies have used quantifying satisfying sexual events as a primary efficacy endpoint in clinical trials which is a capricious outcome due to the lack of standardization between subjects. More quantifiable and patientcentered outcomes like relief of distress, improvement in self-esteem, or interpersonal relationships should be centered and measured. This would require a validated questionnaire that covers all aspects of female sexual function. This questionnaire will need to take into consideration sociocultural expectations of sex and sexuality and can be provided in a variety of languages is also needed.

## Conclusion

HSDD is a common condition that affects millions of people with incidence increasing with age. A comprehensive approach to diagnosis and treatment is key to treatment success. TRT is an effective treatment option for postmenopausal people with HSDD as testosterone is vital to sexual function and health. There is still limited data on the effectiveness in premenopausal people with HSDD. Further research is needed for the long-term effect of TRT in women of all ages.

## Data Availability

All data generated or analyzed during this study are included in this published article.
